# The Role of Adaptive Immunity in Diabetic Retinopathy

**DOI:** 10.3390/jcm11216499

**Published:** 2022-11-02

**Authors:** Mengting Xue, Xiying Mao, Mingkang Chen, Wenjie Yin, Songtao Yuan, Qinghuai Liu

**Affiliations:** Department of Ophthalmology, The First Affiliated Hospital of Nanjing Medical University, Nanjing 210029, China

**Keywords:** adaptive immunity, diabetic retinopathy, lymphocyte

## Abstract

Diabetic retinopathy (DR) is currently one of the common causes of vision loss in working-age adults. It is clinically diagnosed and classified according to the vascular changes in the fundus. However, the activation of immune cells occurs before these vascular changes become detectable. These, together with molecular studies and the positive clinical outcomes of anti-inflammatory treatment, highlight the pivotal involvement of the immune system. The role of innate immunity in DR pathophysiology has been studied in depth, but the contribution of adaptive immunity remains largely elusive. This review aims to summarize our current understanding of the activation mechanism of adaptive immunity in DR microenvironments and to discuss the relationship between adaptive immunity and local vascular units or innate immunity, which opens new avenues for clinical applications in DR treatment.

## 1. Introduction

Diabetic retinopathy (DR) is the most common chronic microvascular complication of uncontrolled diabetes and can lead to preventable blindness. Clinically, DR is currently divided into two stages: non-proliferative diabetic retinopathy (NPDR) and proliferative diabetic retinopathy (PDR). NPDR represents the early stages of DR, and its pathological manifestation involves the loss of pericytes, the formation of acellular capillaries and the thickening of basement membranes [1]. Its molecular mechanism is characterized by the activation of endothelial cells; leukocyte stasis, which further leads to endothelial cell dysfunction; and the decomposition of the blood–retinal barrier (BRB). PDR is the more advanced stage of DR and is characterized by the formation of new blood vessels, resulting in retinal hemorrhage and, eventually, irreversible blindness.

Therefore, the vascular system works as the link between systemic metabolic dysfunction and local lesions. In addition to endothelial cells and pericytes, immune components are important components of the vascular system. Increasingly more studies have shown that the activation of the innate immune system plays an indispensable role in the progression of DR, but the role of the adaptive immune system remains elusive. In this review, we introduce the activation mechanism of the adaptive immune system and focus on the function of adaptive immunity in microangiopathy to determine the potential of immunotherapy in the early intervention of DR.

## 2. Innate Immunity and DR

Innate immunity, also known as congenital immunity or non-specific immunity, is the first line of defense against the invasion of pathogenic microorganisms. Innate immunity in the eye is mainly composed of resident myeloid cells and the complement system. In normal retina, the immune state of the retina is strictly controlled and suppressed. However, a single-cell transcriptome analysis of the retina of nonhuman primates with diabetes showed that cone-secreted TGFβ2 inhibited the activation of microglia [2]. In streptozotocin (STZ)-induced diabetic mice, NLRP3-centered inflammasome was activated in the inner nuclear layer within microglia. Microglial activation was also found in the choroid, retina and fibrovascular membranes (FVMs) of PDR patients [3]. In the single-cell RNA sequencing (scRNA-seq) data of peripheral blood mononuclear cells (PBMC) of patients with diabetic macular edema (DME), CD14++ monocytes (MC) expressing multiple inflammatory cytokines and chemokines were the predominant activated cell population in circulation [4].

The complement system is also an important part of the innate immune system. In the absence of infection, complement proteins circulate in an inactive form. Upon stimulation, the complement system is activated, forming a membrane attack complex (MAC) that can effectively kill cells and producing complement fragments involved in various immune responses [5]. The deposition of MAC was detected in the retinal blood vessels of type 2 diabetic (T2D) patients with a disease course of more than 9 years and in those of DR patients [6,7]. Complement members such as the C5a, C3 and B factors increased in the vitreous humor of PDR patients [8,9,10]. Previous studies demonstrated the positive correlation of serum mannose-binding lectin (MBL), a lectin pathway activator of the complement system, with the development of DR, indicating that MBL is an independent biomarker for DR [11,12].

## 3. Adaptive Immunity and DR

For a long time, immunoregulation in DR has focused on innate immunity, particularly on the microglia, but growing evidence suggests that adaptive immunity is also critical in the development of metabolic inflammation and DR. Adaptive immunity, also known as acquired immunity, can recognize specific pathogenic microorganisms (antigens) or biomolecules. It is primarily made up of T cells (including CD4+ T cells, CD8+ T cells and natural killer T (NKT) cells) and B cells. CD4+ effector T cells can be further subdivided into proinflammatory T helper 1 (Th1), Th17 and Follicular T-helper (Tfh) [13], anti-inflammatory Th2 and Foxp3+ regulatory T (Treg) cells according to their functions and cytokines [14]. CD8+ T cells are essential for adaptive immune responses against infection as they secrete cytokines such as interferon-gamma (IFN-γ) and tumor necrosis factor-alpha (TNF-α) [15]. Balance between Th2 or Tregs, and the effector T cell subsets, such as Th1 or Th17 cells, is critical for immune homeostasis and immune responses.

### 3.1. Changes in Adaptive Immunity during DR: Clinical and Experimental Evidence

Signs of lymphocyte activation were observed in circulation in DR patients, including the reduced surface expression of L-selectin. The reduced expression of L-selectin on CD3+ cells was associated with increased leucocyte adhesion. More CD4+ Tfh cells were found in circulation in DR patients [16,17] and in the lymph nodes and retinal tissues of STZ-induced diabetic mice. The inhibition of Bcl-6, a key transcription factor for Tfh cell development, prevented the up-regulation of Tfh cells and their typical IL-21 cytokines and improved vascular leakage in DR mice or retinal angiogenesis in oxygen-induced retinopathy (OIR) mice [18]. These results suggest the involvement of lymphocyte activation in DR pathogenesis.

In addition, lymphocyte infiltration was also found in the peripheral samples of DR patients. The densities of CD4+ T cells, CD8+ T cells and CD19+ B cells significantly increased in the FVMs of active PDR patients compared with that of epiretinal membranes without DR [19,20]. Marked alterations in the lymphocytes of DR patients indicate the strong correlation of lymphocytes with the occurrence and progression of DR. Therefore, how lymphocytes activate and function becomes questions in the puzzle called DR pathophysiology.

### 3.2. Mechanism of Lymphocyte Activation in Diabetes

Two separate signals are required in the complete activation of lymphocytes. Preliminary antigen-specific signals are sent through antigen receptors: the T-cell receptor (TCR) on T cells and the surface immune globulin (Ig) on B cells. The second signal, called co-stimulation, is independent of antigen receptors and is required for full activation. Well-known costimulatory molecules include B7-1 (CD80) and B7-2 (CD86), expressed on activated antigen-presenting cells (APC). Their ligand, CD28, is correspondently found on the surface of T cells [21].

Obesity is the hallmark of metabolic syndrome and predisposes patients to the development of major chronic metabolic diseases including type 2 diabetes mellitus (T2DM). In adipose tissue, phenotypic changes in T cells, as well as the recruitment of B and T cells, preceded the infiltration of macrophages [22]. A study demonstrated that patients with diabetic ketoacidosis (DKA) and hyperglycemia exhibited increased levels of proinflammatory cytokines and activated CD4+ and CD8+ T lymphocytes [23]. In in vitro studies, the expression of CD28-like costimulatory molecule ICOS (CD278) on the surface of T cells was induced by high glucose (HG) levels or advanced glycation end products (AGEs). Higher levels of IFN-γ, interleukin-4 (IL-4), IL-10 and other cytokines were found in the supernatant when T cells were co-cultured with ICOSL-expressing human umbilical vein endothelial cells (HUVECs) but were not observed in non-contact T cells or T cells treated with HUVEC conditioned medium [24]. This indicates that direct cell–cell contact between T lymphocytes and endothelial cells is necessary for T cell activation in HG or AGE treatment.

Evidence that elevated major histocompatibility complex class II (MHC II) in insulin-resistant tissues is involved in the activation of CD4+ T cells is mounting. A study by Shirakawa et al. demonstrated that activated CD4+ T cells increased in the visceral adipose tissue of obese mice [25]. These cells, called adipocyte resident T cells (ART), regulated the metabolic and inflammatory response, mainly through the up-regulation of MHC II and the secretion of leptin by adipocytes to activate CD4+ T cells and subsequently triggered adipose tissue inflammation. MHCII−/− mice fed a high fat diet (HFD) had less adipose inflammation and insulin resistance than wild-type mice [26]. These studies suggest that CD4+T, which plays an important role in inflammation and insulin resistance, can be activated by adipocytes in an antigen-specific, contact-dependent direct pathway.

In the diabetic state, low levels of effective insulin concentrations as well as high contents of glucose and free fatty acids provide an environment for oxidative stress and inflammatory pathway activation [27]. High levels of glucose can form intracellular oxidants in lymphocytes, activating kinase phosphorylates such as IκB, which release nuclear factor-kappaB (NF-κB) into the nucleus. NF-κB binds to κB binding sites in the promoter region of inflammatory genes, leading to the transcription and translation of several proinflammatory mediators [28]. Proinflammatory mediators, such as cytokines, adhesion molecules (intercellular adhesion molecule (ICAM) and platelet endothelial cell adhesion molecule (PECAM)), prostaglandins and arachidonic acid, subsequently lead to the activation of T lymphocytes.

**Figure 1:**
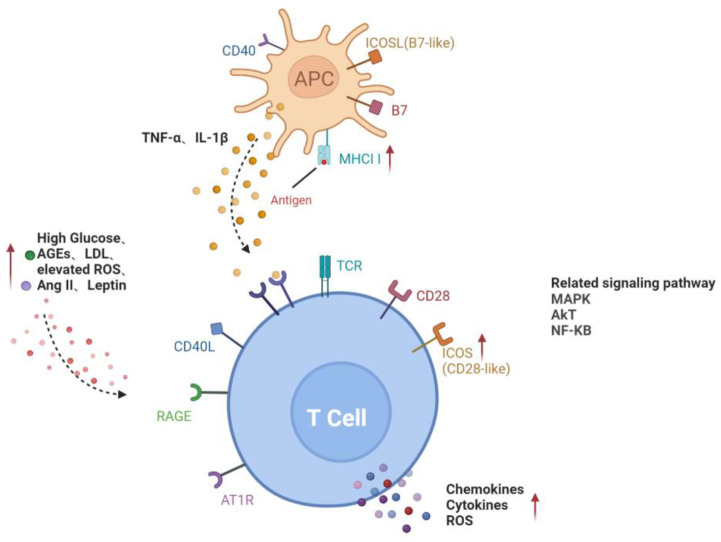
Activation pathways of T lymphocytes in diabetes. Direct contact between APC and T cells through MHCII and co-stimulators on the cell surface; increased glucose, AGEs, LDL, ROS, Ang II and leptin in diabetic microenvironment or cytokines such as TNF-α and IL-1β secreted by APC activate T cells, forming the paracrine pathway. Red arrows represent the upregulation of gene expression or increase of risk factors in diabetes. Black dotted arrows stand for cellular interaction through paracrine pathway. AGEs, advanced glycation end-products; AT1R, angiotensin II type 1 receptor; ICAM-1, intercellular adhesion molecule-1; LDL, low-density lipoprotein; RAGE, receptor for advanced glycation end products; ROS, reactive oxygen species; ICOSL, inducible co-stimulatory molecule ligand; MHCII, major histocompatibility complex class II; MAPK, mitogen-activated protein kinase; AKT, v-akt murine thymoma viral oncogene homologue; NF-KB, nuclear factor-kappaB.

In conclusion, the activation of lymphocytes in DR is mainly involved in two pathways, including a direct contact pathway (up-regulated MHC II molecules or co-stimulators) and an indirect paracrine pathway (a higher level of cytokines), in a diabetic environment (Figure 1).

### 3.3. Interaction between Lymphocytes and Retinal Vascular Unit in DR

Retinal vascular dysfunction can be caused by a variety of factors, such as AGE products and receptors, proinflammatory cytokines and chemokines, proliferator-activated receptor-γ disruption, growth factors, oxidative stress and microRNA [29,30,31,32,33,34,35]. These factors promote retinal endothelial apoptosis and neovascularization, leading to the development of DR. Lymphocyte infiltration also contributes to the formation of prooxidative and proinflammatory microenvironments, which cannot be neglected in the progression of diabetic microangiopathy.

Lymphocytes affect target cells by secreting inflammatory cytokines. Once activated, Th1 cells release large amounts of cytokines such as IFN-γ, IL-2 and TNF, thereby triggering cell-mediated immune response and phagocyte-dependent inflammation [18]. Increased levels of IFN-γ and IL-2 have been observed in the vitreous or aqueous humor of patients with diabetes or DR [36,37] and in retina of diabetic rats [38].

Endothelial barrier function is tightly controlled by a wide range of signaling cascades, including the nitric oxide (NO)/cyclic guanosine monophosphate (GMP) pathway. IFN-γ was also demonstrated to promote the permeability of HUVECs, which may be associated with an increase in NO production by IFN-γ [39]. In addition, IFN-γ induced disruptions in the endothelial cell junction and reduced the endothelial barrier properties via Rho kinase (ROCK)-mediated cytoskeleton contraction. The neutralization of IFN-γ attenuated vascular ROCK activity and, therefore, improved the endothelial barrier integrity and pericyte coverage [40]. The level of TNF-α significantly increased in DR patients, as shown in a meta-analysis study [41]. TNF-α reduced the expression of tight junction proteins by activating protein kinase C zeta (PKCζ) and NF-κB, thereby increasing the permeability of retinal endothelial cells [42].

**Figure 2:**
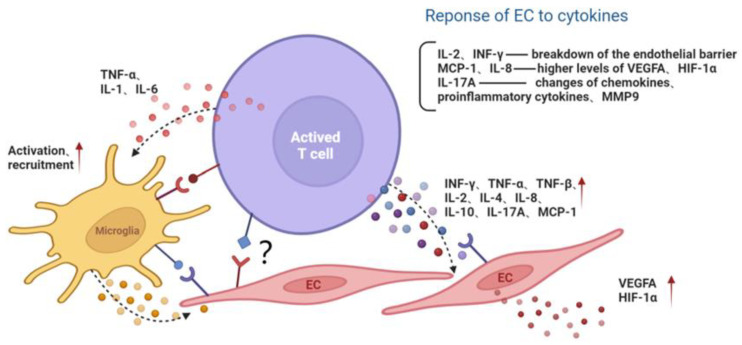
The interaction among activated T lymphocyte, endothelial cell and microglia. Activated T cells secrete cytokines or directly contact microglia and the EC, aggravating the inflammatory microenvironment and endothelial dysfunction. Red arrows represent the upregulation of gene expression. Black dotted arrows stand for cellular interaction through paracrine pathway. EC, endothelial cell; MMP9, matrix metalloproteinase 9; MCP-1, monocyte chemoattractant protein-1; INF, interferon; TNF, tumor necrosis factor; IL, interleukin; VEGFA, vascular endothelial growth factor A; HIF-1α, hypoxia inducible factor-1α.

In addition, the proinflammatory cytokine IL-17A, mainly produced by Th17 cells, may participate in the development of retinal inflammation and long-term vascular pathology in diabetic mice. T cells and neutrophils expressing IL-17A were found in diabetic retinal vasculature. Furthermore, the IL-17A receptor was expressed on Müller glia cells, retinal endothelial cells and photoreceptors [43]. IL-17A signaling induced photoreceptor cells to produce ROS, and systemic ablation of IL-17A reduced retinal inflammation, oxidative stress and vascular leakage. In addition, IL-17A ablation was related to significant reductions in the levels of chemokines (CCL2, CCL5, CX3CL1, CXCL1 and CXCL5), proinflammatory cytokines (TNF and IL-1) and growth factors (G-CSF and GM-CSF) and to increases in the levels of the matrix metalloproteinase 9 (MMP-9) inhibitor and the tissue inhibitor of metalloproteinase (TIMP-1) [43]. Among them, MMP-9 is related to the release of TGF-β1 and VEGF and can participate in the destruction of the blood–retina barrier (BRB), inflammation, angiogenesis, mitochondrial damage and apoptosis by changing the vascular permeability [44] (Figure 2).

Endothelial cells can be protected by inhibiting the activation of T lymphocytes and the paracrine effect of T cells. It was found that after blocking the ICOS/ICOSL pathway with the anti-ICOS antagonist antibody [24] or knocking down Ras guanine nucleotide releasing protein 4 (RasGRP4), the infiltration of CD3+ T lymphocytes and F4/80+ macrophages was reduced. The secretion of inflammatory cytokines such as IL-6, TNF-α, ICAM-1 and vascular cell adhesion molecule-1 (VCAM-1) were inhibited, along with the sustained viability of endothelial cells in diabetic mice [45].

Direct contact of activated T cells with vasculature target cells can also play a pathogenic role. The co-culture of CD8+ T cells with endothelial cells displayed more striking damage on endothelial viability compared to the treatment of the conditioned media of CD8+ T cells derived from ischemic tissues of T2D patients and a T2D Lepr^db/db^ mouse model, indicating the nonredundant role of the direct contact of lymphocyte with endothelial cells [46]. However, the precise mechanism remains to be further investigated.

### 3.4. The Interaction of Lymphocytes with DR Resident Innate Immunity

Microglia are important components of retinal innate immunity. Adaptive immunity plays a vital role in the development and activation of microglia. Single-cell sequencing of the central nervous system revealed that, in the absence of murine CD4+ T cells, resident microglia remained in the fetal state. The final stage of normal microglial differentiation was defective in MHC II KO mice [47]. It seems that CD4+T cells affect the development of microglia partially due to their direct contact. In addition to normal development, adaptive immunity is also involved in microglial activation under pathological circumstances.

Under a high-glucose environment, AGE contributes to Th1 cell activation and its production of IFN-γ. IFN-γ in turn promotes M1 polarization and enhances its proinflammatory function by inducing the release of IL-1, IL-6 and TNF-α. In contrast, Th2 cells can produce anti-inflammatory IL-4 and IL-13, thereby causing macrophages to differentiate into M2 [48,49]. The imbalance in macrophage polarization between proinflammatory M1 and anti-inflammatory M2 is regarded as a key factor in the development of obesity and T2DM [50]. In diet-induced obesity, CD8+ T cells in adipose tissue increased and induced the recruitment and differentiation of M1, which secreted TNF-α, whereas the amount of M2, which secreted IL-10, decreased in adipose tissue [51]. Thus, activated Th1 and Th2 induce M1/M2 polarization in a paracrine manner. Studies showed that CD8+T could stimulate the transformation from M2 to M1 and could aggravate inflammation and insulin resistance. The elimination of CD8+ T inhibited macrophage recruitment, attenuating insulin resistance and inflammation [52]. Therefore, manipulating microglia into an M2-polarized state provides new insights into targeting neuroinflammation and may be a therapeutic strategy to delay and/or prevent the deterioration of visual function in diabetic patients [53,54].

It was been revealed that treatment with CD28 super-agonists (CD28SAs) increased the amount of circulating Tregs and upregulated M2 dominant macrophage/microglia expression in T2DM stroke mice. In addition, the adoptive transfer or anti-IL-2-induced expansion of Tregs reduced the activation of Tmem119+ retinal microglia and further reduced retinal vascular occlusion and neovascularization. The upregulation of costimulatory molecules and proinflammatory mediators was attenuated by CTLA-4 blockade [55]. Thus, Tregs may play a potential protective role in the pathogenesis of DR, which serves as a potential intervention of DR. However, the activation state of microglia dynamically changed in a multi-facet disease setting. Treg cells transform into proinflammatory Th17-like cells in T2D patients. The immunosuppressive activity of Treg cells was negatively correlated with body mass index (BMI) and HbA1c levels [56]. Therefore, an increase in Tregs or the conversion of local proinflammatory T cells to anti-inflammatory Tregs may be considered a long-term and sustained therapeutic strategy for DR treatment.

## 4. Conclusions and Perspectives: Potential Therapeutic Strategies

Current treatments for DR include strict control of the systemic condition, intravitreal drug therapy, laser photocoagulation and surgery. DR is a complex neurovascular disease that affects not only the vascular structure but also the nerve tissue of the retina. The activation of immune cells occurs earlier than neural degradation and the consequential microvascular abnormalities. The pathological interaction among neurons, vascular cells, and local immune cells that, together, form neurovascular units is thought to be related to the early progression of DR. Targeting lymphocyte-mediated microglial polarization and lymphocyte polarization may be a novel strategy to modulate neurovascular inflammation in DR.

Interference with cytokines produced by lymphocytes exhibits a broad therapeutic prospect in DR. Adalimumab is the first fully human monoclonal antibody directed against TNF that is approved in December 2002 by the US Food and Drug Administration (FDA) for the treatment of moderate to severe rheumatoid arthritis (RA) [57]. Adalimumab has also shown efficacy in treating refractory uveitis in multiple settings. Studies showed that Adalimumab induced a significant reduction in microglial activation and the reversion of M1/M2 polarization, resulting in photoreceptor survival in retinal degeneration [58]. Moreover, significant increases in the number and function of Treg cells were observed in RA patients with Adalimumab [59]. It is tempting to speculate that Adalimumab could be a promising therapy for DR, attributed to its potential function of immunosuppression and neuroprotection.

The regulation of T cell polarization also serves as an alternative strategy for modulating vascular inflammation, including the balance of Th1/Th2 and Th17/Treg polarization. In a prospective open-label, phase 1, phase 2a study, low-dose IL-2 treatment was found to be able to promote the survival and immunosuppressive function of Treg and clinical improvements in patients with autoimmune vasculitis [60]. Interestingly, the usage of the humanized anti-IL-6 receptor antibody Tocilizumab in the treatment of RA showed that IL-6 blockade will skew the polarization from Th17 to Treg cells in favor of anti-inflammatory responses [61].

Furthermore, circulating leukocytes, including lymphocytes, serve as a messenger that transmits systemic pathological information to peripheral tissue. Thus, targeting lymphocytes can be considered in early interventions for DR, a common ocular diabetic complication. Above all, the modulation or blockade of lymphocyte activation may be of interest in developing a general therapeutic strategy to prevent systemic complications, other than DR, in diabetic patients.

## Data Availability

Not applicable.
